# *In vitro* pharmacological profiling of ITE amide bioisosteric aryl hydrocarbon receptor agonist

**DOI:** 10.3389/fphar.2026.1931941

**Published:** 2026-10-02

**Authors:** Zdeněk Dvořák, Aneta Grycová, Radim Vrzal, Miroslav Soural, Iveta Zůvalová, Jakub Přikryl

**Affiliations:** 1 Department of Cell Biology and Genetics, Faculty of Science, Palacký University, Olomouc, Czechia; 2 Department of Organic Chemistry, Faculty of Science, Palacký University, Olomouc, Czechia

**Keywords:** AhR receptor, bioisostere, ITE, pharmacological profiling, structure optimization

## Abstract

Previously, we reported ITE-derived bioisostere 2-(1*H*-indole-3-carbonyl)-N-methyl thiazole-4-carboxamide (ITE-CONHCH_3_) as a high-affinity ligand and potent agonist of the aryl hydrocarbon receptor (AhR) with anti-colitis properties. In the current study, we present its initial *in vitro* pharmacological profiling. Physicochemical analysis revealed low aqueous solubility (22 µM), excellent chemical stability in human and mouse plasma, and strong plasma protein binding (>98%). Off-target screening against steroid and nuclear receptors confirmed high AhR selectivity. In Caco-2 cells, ITE-CONHCH_3_ exhibited high permeability with no active efflux. Interestingly, the compound partitioned substantially into mouse red blood cells (47%) but only weakly into human RBCs (16%). In reporter gene assays, ITE-CONHCH_3_ displayed ∼40-fold higher agonist potency in human cells compared to mouse cells. Its ability to activate AhR was further validated by a cell-free AhR-ARNT heterodimerization assay and by the induction of canonical (*CYP1A1*) and non-canonical (*PAI-1*) genes expression in Caco-2 cells. In conclusion, ITE-CONHCH_3_ possesses a favorable pharmacological profile supporting its potential preclinical development. However, the observed differences in RBC partitioning and AhR potency between species must be considered when interpreting mouse-derived data.

## Introduction

The aryl hydrocarbon receptor (AhR) is a ligand-activated transcription factor belonging to the basic helix-loop-helix-PAS family of proteins. Originally recognized for its role in xenobiotic metabolism, the AhR is now understood to play a pivotal role in numerous physiological processes, including immune homeostasis, cell cycle regulation, and epithelial barrier integrity. Consequently, the dysregulation of AhR signaling has been implicated in several pathophysiologies and diseases (Coumoul et al., 2026). This has positioned the AhR as a promising therapeutic target for inflammatory conditions (e.g., inflammatory bowel disease, atopic dermatitis, and psoriasis), viral infections (e.g., SARS-CoV-2), and various malignancies, such as head and neck cancer and leukemia (Dvorak et al., 2020a; Polonio et al., 2025; Silverberg et al., 2024; Pinto et al., 2023; Grycova et al., 2025; Barreira-Silva et al., 2025). In terms of pharmacological activity, therapeutic AhR ligands exhibit high degree of complexity, acting as either agonists or antagonists, depending on the cellular context. AhR agonists typically exert anti-inflammatory effects, and the concept of functional selectivity (agonist bias) suggests that the health outcomes of AhR activation - whether beneficial or detrimental–are highly dependent on the specific chemical scaffold and binding affinity of the ligand. A structurally prominent group of AhR ligands consists of indoles, which include natural and dietary compounds, synthetic compounds, drugs, endogenous compounds and microbial metabolites (Dvorak et al., 2021). Notably, microbially-derived indoles with substitutions at the C3 position have been identified as key signaling molecules in the human gut and skin, where they mediate health-promoting effects through AhR activation (Zelante et al., 2013; Roager and Licht, 2018; Vyhlidalova et al., 2020).

One such molecule, a high affinity ligand and potent AhR agonist ITE (methyl-2-(1*H*-indole-3-carbonyl)thiazole-4-carboxylate), was initially isolated from porcine lungs and proposed as a potential endogenous human metabolite (Song et al., 2002). However, subsequent studies questioned its endogenous origin in humans, suggesting instead that it is produced by *Thermosporothrix hasakensis*, a bacterial endosymbiont found in pig lungs (Sladekova et al., 2023; Grycova et al., 2022; Park et al., 2015). The primary barrier to the clinical translation of ITE is its extreme chemical instability. Chen *et al* reported a half-life of only ≈44 min in mouse plasma (Chen et al., 2020). Our previous research confirmed that approximately 95% of ITE is degraded within 1 hour in mammalian cell cultures, primarily due to the rapid cleavage of its ester bond, yielding a carboxylic acid derivative with only marginal AhR activity (Grycova et al., 2022). To address this limitation, we synthesized a methyl-amide bioisostere (ITE-CONHCH_3_). This modification strongly enhanced the compound´s chemical stability while preserving robust AhR potency, although slightly lower than that of the parent ITE. We further validated its therapeutic potential by demonstrating significant anti-colitis effects in DSS-induced acute colitis mouse model (Grycova et al., 2022).

In the present study, we conducted a comprehensive *in vitro* pharmacological profiling of ITE-CONHCH_3_. Our results demonstrate that this bioisostere possesses an excellent stability profile in both human and mouse plasma, high plasma protein binding, and high AhR selectivity. Notably, the compound exhibits high intestinal permeability with no detectable active efflux, suggesting favorable oral bioavailability. A particularly intriguing finding is the species-specific potency: ITE-CONHCH_3_ displayed substantially higher agonist activity in human cells compared to mouse cells. Mechanistically, ITE-CONHCH_3_ strongly promoted AhR-ARNT dimerization in cell-free assays and effectively induced both canonical (*CYP1A1*) and non-canonical (*PAI-1*) gene expression in human colon adenocarcinoma line Caco-2 that structurally and functionally mimics the human intestinal epithelium. Taken together, the favorable pharmacological profile of ITE-CONHCH_3_ identifies it as a superior candidate for further preclinical and clinical development in the treatment of AhR-mediated diseases.

## Materials and methods

### Chemicals

The synthesis of ITE-CONHCH_3_ was performed as described previously (Grycova et al., 2022; Kwon et al., 2015), and the purity of the ITE-CONHCH_3_ preparation was >95%, as assessed by HPLC analyses. Dimethylsulfoxide (DMSO; cat# D8418; purity >99.9%) and 6-Formylindolo[3,2-*b*]carbazole (FICZ; cat# SML1489; purity >95% by HPLC) were purchased from Sigma Aldrich–Merck. TNFα (cat# 300-01A; purity >98%), IFNγ (cat# 300–02; purity >98%), and IL-1β (cat# 200-01B; purity >98%) were purchased from PreproTech and their purity and identity were determined by SDS-PAGE electrophoresis and HPLC analyses.

### Thermodynamic aqueous solubility

Solubility was determined in PBS (pH 7.4) at room temperature using the shake-flask method as described elsewhere (Lipinski et al., 2001). The concentration of ITE-CONHCH_3_ was quantified by HPLC. Amiodarone and propranolol were used as reference compounds.

### Plasma stability

ITE-CONHCH_3_ (2 μM) was incubated at 37 °C in K_2_EDTA-stabilized human or mouse plasma for 0, 10, 20, 30, 60, and 120 min. Propantheline (2 μM) served as a reference compound that undergoes degradation in plasma.

### Plasma protein binding

A binding of ITE-CONHCH_3_ (2 μM) and reference compounds (amiodarone, propranolol, warfarin) to human and mouse plasma proteins, as well as human liver microsomal proteins, was studied using an ultracentrifugation technique (2.5 h; 37 °C) (Nakai et al., 2004; Obach, 1997). The HPLC peak areas of ITE-CONHCH_3_ in the supernatant (free) and protein matrix (bound) were used to calculate the percentage of binding according to the formula:

$$\text{binding }\%=100\times \left(1-c_{\text{free}}/\left(c_{\text{bound}}+c_{\text{free}}\right)\right).$$

### Red blood cell partitioning

The assay was performed according to standard protocols (Hinderling, 1997; Yu et al., 2005). Briefly, ITE-CONHCH_3_ (2 μM) or methazolamide (2 μM) were incubated in whole blood for 1 h at 37 °C. Calculations were conducted using the following parameters (PL = plasma; H = hematocrit; WB = whole blood; C = concentration):

$$Adjusted\;Ratio,\text{accounting for hematocrit}:\left(C_{\text{spiked}\;\text{PL}}\;/\;C_{\text{PL}\;\text{from}\;\text{spiked}\;\text{WB}}\right)\times 1/\left(1-H\right)$$

$$Partition\;coefficient:K_{p\left(\text{RBC}/\text{PL}\right)}=\left[\left(C_{\text{spiked}\;\text{PL}}\;/\;C_{\text{PL}\;\text{from}\;\text{spiked}\;\text{WB}}\right)-1\right]\times \left(1/H\right)+1$$

*Percentage partitioned* represents the fraction of the total compound sequestered from plasma phase in spiked whole blood. Red blood cell partitioning was considered positive if the Adjusted Ratio was >1.0, and if K_p(RBC/PL)_ was >0.

### Permeability assay

The permeability assays were performed using Caco-2 cells monolayers at 37 °C (Hidalgo et al., 1989): Transport was measured in both apical-to-basolateral (A–B; 60 min) and basolateral-to-apical (B–A; 40 min) directions. Concentrations of the test compounds were determined by HPLC-MS/MS. The ***apparent permeability coefficient (P***_***app***_ ***)*** was calculated using the following equation:

$P_{\text{app}}\;\left(\text{cm}.s^{-1}\right)=V_{R}\times C_{R,\text{end}}\;/\;\Delta t\times A\times \left(C_{D,\text{mid}}\;–\;C_{R,\text{mid}}\right),$ where **V**_**R**_ is the receiver chamber volume; **C**_**R,end**_ is the concentration in the receiver chamber at the final time point, **Δt** is the incubation time, and **A** is the surface area of the cell monolayer. **C**_**D,mid**_ represents the calculated mid-point concentration in the donor chamber (mean of concentrations at **t = 0** and **t**_**end**_), and **C**_**R,mid**_ is the mid-point concentration in the receiver chamber (half of **C**_**R,end**_). The ***recovery*** of the test compounds was calculated as follows:

$\text{Recovery }\%=100\times \left(V_{D}\times C_{D,\text{end}}+V_{R}\times C_{R,\text{end}}\right)/V_{D}\times C_{D0},$ where V_D_ and V_R_ are the volumes of the donor and receiver chambers, **C**_**D,end**_ and **C**_**R,end**_ are the final concentrations in the respective chambers, and **C**_**D0**_ is the initial donor concentration. To ensure monolayer integrity, fluorescein permeability was assessed (A-B direction, pH 7.4) immediately following the assay. Only monolayers with a fluorescein permeability <1.5 × 10^−6^ cm.s^-1^ were included in the study.

### Reporter gene assay

The stably transfected human reporter cell lines AZ-AHR (Novotna et al., 2011), LS174T-AHR-luc (Vrzalova et al., 2022), AZ-GR (Novotna et al., 2012), AIZ-AR (Bartonkova et al., 2015), PZ-TR (Illes et al., 2015), and IZ-VDRE (Bartonkova et al., 2016) were used for the evaluation of the transcriptional activity of aryl hydrocarbon receptor (AhR), glucocorticoid receptor (GR), androgen receptor (AR), thyroid hormone receptor, and vitamin D receptor (VDR), respectively. The activity of mouse AhR was studied in AheRePa reporter cells derived from mouse hepatoma Hepa1c1c7 cells (Vrzal et al., 2023). Following incubation with tested compounds, the cells were lysed and luciferase activity was measured on a Tecan Infinite M200 Pro plate reader (Schoeller Instruments, Czech Republic).

### AhR-ARNT heterodimerization assay in rabbit reticulocyte lysates

The full-length wild-type human AhR ORF sequence (GenBank accession no. NM_001621.5) was inserted into the expression vector pcDNA3.1(+)-C-6His *via* HindIII and NotI restriction sites (termed FL-WT-hAHR-C-6His). The native stop codon was removed to allow in-frame fusion with the vector-encoded His tag; the NotI cloning site introduces a short alanine linker (Ala-Ala) between the hAHR coding sequence and the 6×His tag, as inherent to the pcDNA3.1(+)-C-6His vector design. The full-length wild-type human ARNT ORF sequence (GenBank accession no. NM_001668.4) was inserted into the expression vector pcDNA3.1(+) *via* HindIII and BamHI restriction sites (termed FL-WT-hARNT). Both target proteins were co-expressed using the TnT® Quick Coupled Transcription/Translation System (L1170, Promega) in the presence of tested compounds, a vehicle (0.1% v/v DMSO) or the model AhR agonist FICZ (100 nM) for 60 min at 30 °C. Following incubation, protein heterodimers were purified using HisPur™ Cobalt Superflow Agarose (Thermo Scientific) and eluted with a buffer containing 20 mM HEPES, 300 mM NaCl, and 300 mM imidazole (pH 7.4). Eluates were concentrated and the elution buffer was replaced with an equilibration/wash buffer (20 mM HEPES, 300 mM NaCl, 10 mM imidazole, pH 7.4) using 30 kDa Amicon® Ultra-0.5 centrifugal filters (Merck). Both eluates and input samples (taken prior to His-tag purification) were resolved by standard SDS-PAGE followed by Western blot analysis. Immunodetection was performed using the following antibodies: AhR (mouse monoclonal, sc-133088, A-3, Santa Cruz Biotechnology), ARNT1 (mouse monoclonal, sc-17812, G-3, Santa Cruz Biotechnology), and a horseradish peroxidase-conjugated anti-mouse secondary antibody (7076S, Cell Signaling Technology). The degree of AhR-ARNT complex formation was calculated from band intensities, normalized to the AhR signal in the eluate, and expressed as fold induction over the vehicle control.

### qRT-PCR

Caco-2 cells (ECACC 86010202; passage 22–25) were seeded into 12-well plates at a density of 2 ×10^5^ cells *per* well in 1 mL of culture medium and cultured for 20–21 days to reach optimal differentiation. Following differentiation, cells were treated for 4 and 24 h with ITE-CONHCH_3_, vehicle (DMSO, 0.1% v/v), or FICZ, either alone or in combination with a cytokine cocktail (“MIX”) containing 20 ng/mL TNF-α, 10 ng/mL IL-1β, and 10 ng/mL IFN-γ. Total RNA was isolated using TRI Reagent®, and reverse transcription was performed with M-MuLV Reverse Transcriptase. The mRNA levels of *CYP1A1, PAI-1* and *GAPDH* were determined using the KiCqStart Probe Assay (Sigma Aldrich, Prague, Czech Republic) with the following cycling conditions: an activation at 95 °C for 20 s, followed by 45 cycles of denaturation at 95 °C for 5 s and annealing/elongation at 58 °C for 30 s (Table 1). Expression of *TNFα* (Assay ID: Hs99999043_m1), *CXCL8* (IL-8; Assay ID: Hs00174103_m1), and *IL-1β* (Assay ID: Hs01555410_m1) was quantified using TaqMan gene expression assay (FAM) (ThermoFisher Scientific). The program consisted of activation at 95 °C for 20 s, followed by 40 cycles of denaturation at 95 °C for 1 s and annealing/elongation at 60 °C for 20 s. All analyses were conducted using a Light Cycler 480 II (Roche Diagnostic Corporation, Prague, Czech Republic). Measurements were performed in triplicate, and gene expression was normalized to *GAPDH* as a housekeeping gene. The data were processed by the delta-delta method.

**TABLE 1:** Probes used in qRT-PCR.

Name	Oligo type	5'Fluorophore	Sequence (5' to 3')	3'-Quencher
GAPDH	Hydrolysis probe	FAM	ACT​AAC​CCT​GCG​CTC​CTG​CCT​CGA​T	OQA
GAPDH F	Primer		GAA​GGA​AAT​GAA​TGG​GCA​GC	
GAPDH R	Primer		TCT​AGG​AAA​AGC​ATC​ACC​CG	
CYP1A1	Hydrolysis probe	FAM	GCA​ACG​GGT​GGA​ATT​CAG​CGT​GCC​A	OQA
CYP1A1 F	Primer		GGA​AGT​GTA​TCG​GTG​AGA​CC	
CYP1A1 R	Primer		CAT​AGA​TGG​GGG​TCA​TGT​CC	
PAI-1	Probe	FAM	GTG​GCC​TCC​TCA​TCC​ACA​GCT​GTC​A	OQA
PAI-1 F	Primer		GCT​GCA​GAA​AGT​GAA​GAT​C	
PAI-1 R	Primer		CTC​CAT​GAT​GAT​CTC​CTC​GG	

## Results

### Physicochemical properties and plasma stability of ITE-CONHCH_3_

The initial profiling of ITE-CONHCH_3_ (Figure 1A) revealed a rather low thermodynamic aqueous solubility of 22.4 µM (Figure 1B), which, while limited, was much higher than the reference compound amiodarone. Stability assays demonstrated that the compound is highly stable in both human and mouse plasma over 120 min, contrasting with the rapid degradation observed for propantheline (Figure 1C). These data corroborate our previous findings showing that the stability of ITE-CONHCH_3_ in cell cultures is superior to parental ITE compound (Grycova et al., 2022). Furthermore, ITE-CONHCH_3_ showed strong plasma protein binding (>98%) across species (Figure 1D).

**FIGURE 1:**
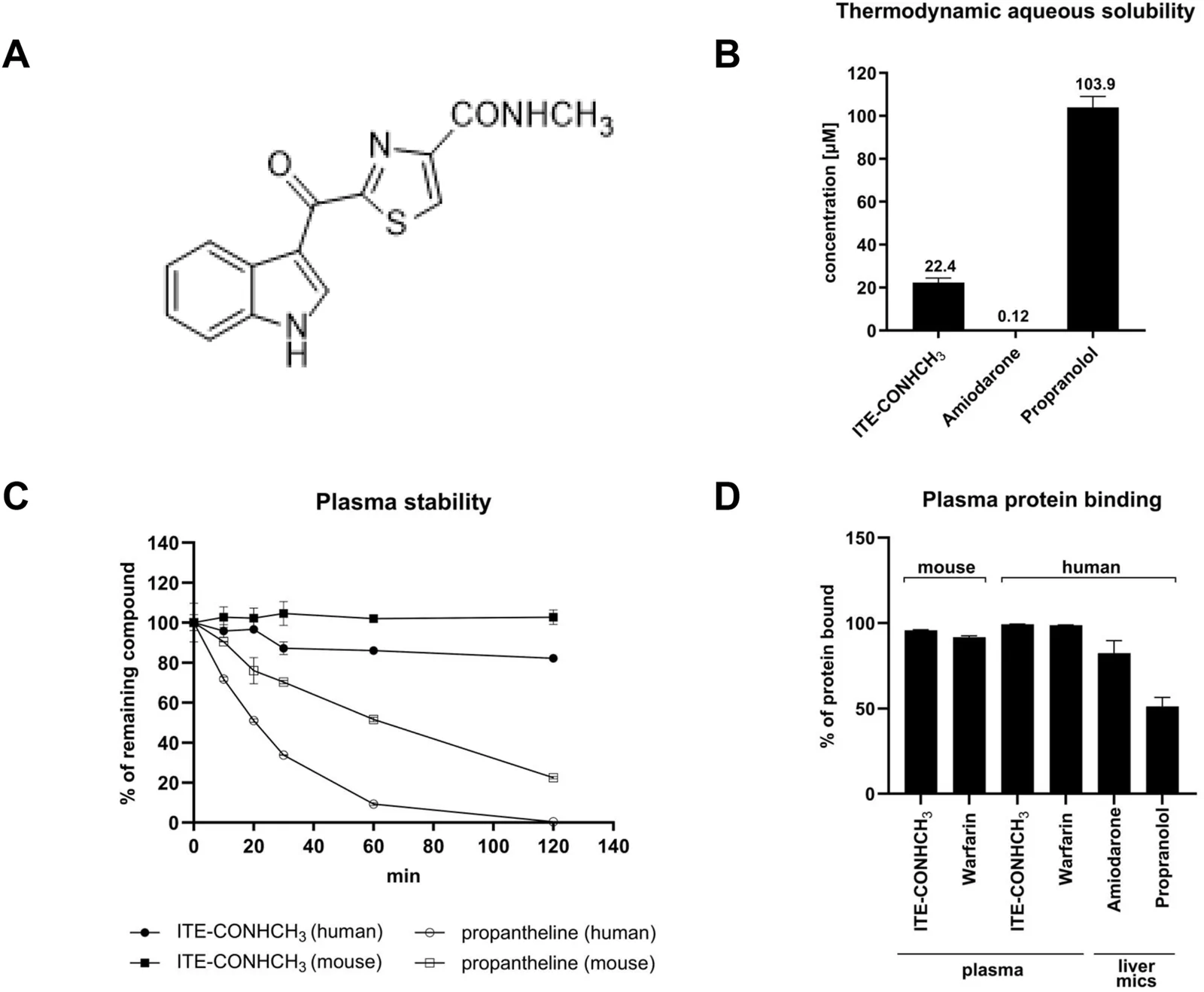
Physicochemical analysis of ITE-CONHCH_3_. **(A)** Chemical structure of 2-(1*H*-indole-3-carbonyl)-N-methyl thiazole-4-carboxamide (ITE-CONHCH_3_). **(B)** Thermodynamic aqueous solubility: Incubations were performed in PBS (pH 7) at room temperature. Data are presented as mean ± SD (n = 3). **(C)** Plasma stability: Stability of ITE-CONHCH_3_ (2 μM) and the reference compound propantheline was in human and mouse plasma over incubation times up to 120 min. The data are mean ± SD (n = 2). **(D)** Binding to plasma proteins: Binding of ITE-CONHCH_3_ and reference compounds to human and mouse plasma proteins, as well as human liver microsomal proteins (“liver mics”), determined by ultracentrifugation. Data are mean ± SD (n = 3). The final DMSO concentration was 0.1% v/v in all incubation assays.

### Cellular uptake and intestinal permeability

Blood distribution studies uncovered notable species-specific differences in red blood cell (RBC) partitioning. While sequestration into human RBCs was low (16.8%), a much higher partitioning was observed in mouse blood (46.9%; Figure 2A). In the Caco-2 cell model, ITE-CONHCH_3_ exhibited high apical-to-basolateral permeability (P_app_ ≈ 21 × 10^−6^ cm/s). This permeability was not substantially altered by the efflux pump inhibitor zosuquidar, suggesting that the compound is not a substrate for active efflux transporters (Figure 2B).

**FIGURE 2:**
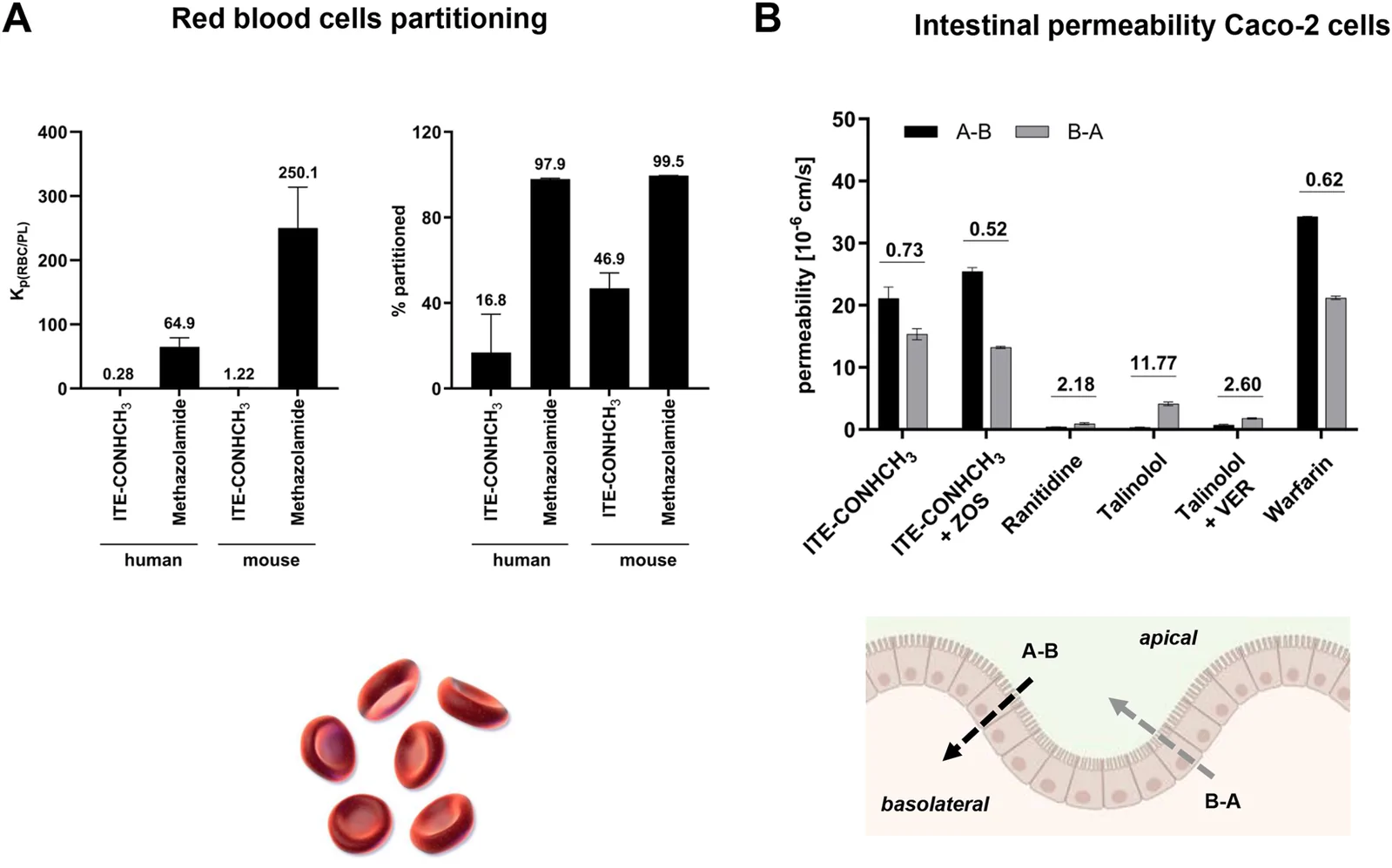
*In vitro* cellular uptake of ITE-CONHCH_3_. **(A)** Red blood cells partitioning: Incubations with ITE-CONHCH_3_ or methazolamide (both at 2 μM) were performed for 1 h at 37 °C. Red blood cell partition coefficient (K_p(RBC/PL)_) was calculated as described in the Methods section. Data represent the mean of triplicate and duplicate measurements for ITE-CONHCH_3_ and methazolamide, respectively. **(B)** Intestinal permeability in Caco-2 cells: Assays with ITE-CONHCH_3_ (10 μM) and reference compounds were carried out at 37 °C, with incubation times of 60 min **(A,B)** and 40 min **(A,B)** The data are presented as mean ± SD (n = 3). Efflux ratios are shown in the inset.

### Selectivity against nuclear and steroid receptors

To assess off-target activity, ITE-CONHCH_3_ was screened against a panel of human receptors (TR, VDR, AR, and GR). The compound showed no agonistic activity for any of the tested receptors up to a concentration of 10 µM (Figure 3). In antagonist mode, a slight concentration-dependent decrease in AR and GR activity was noted; however, the overall results, together with previous findings (Grycova et al., 2022) confirm a high selectivity for the AhR.

**FIGURE 3:**
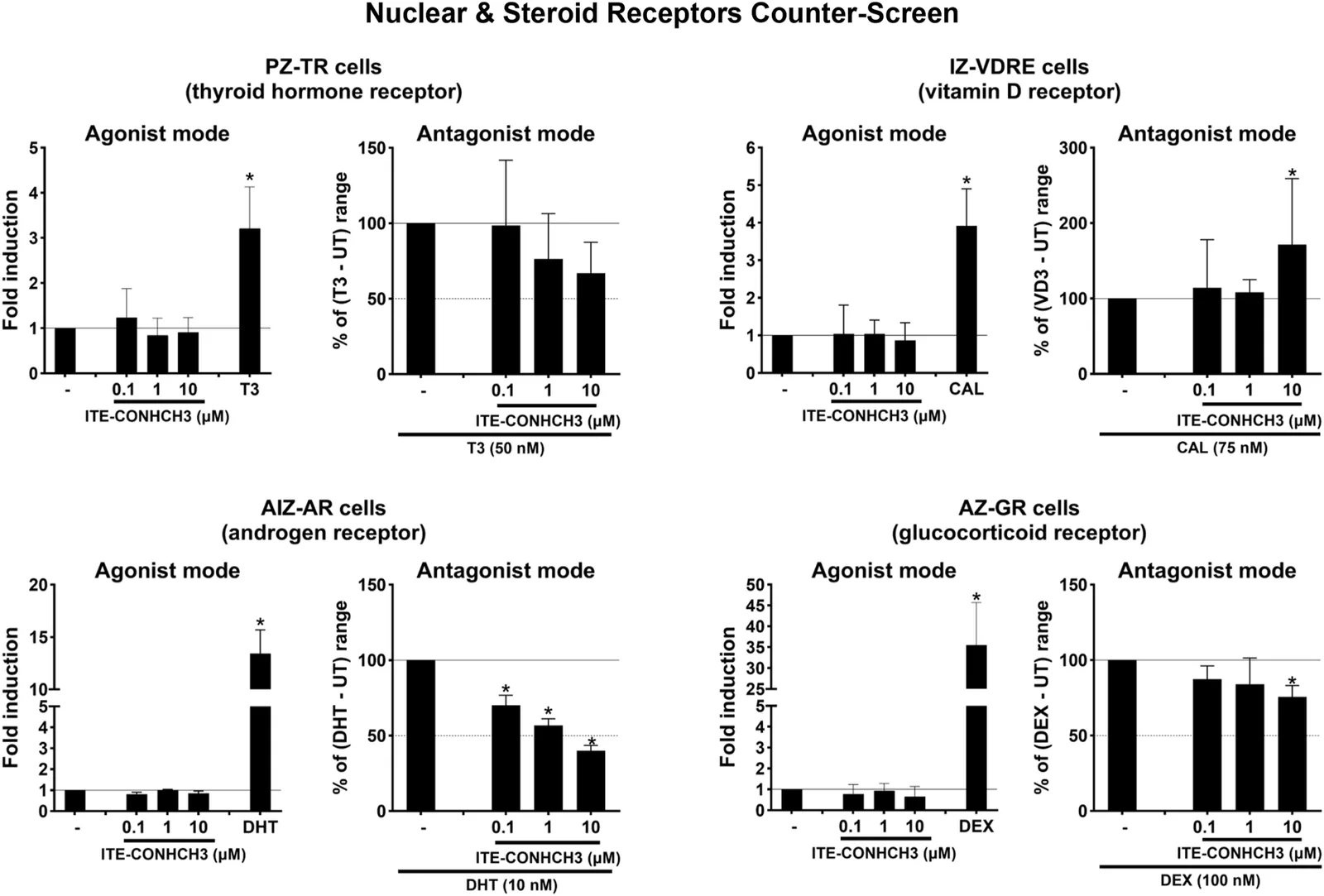
Screening of ITE-CONHCH_3_ against nuclear and steroid receptors. Assays were performed in stably transfected reporter gene cell lines: PZ-TR (thyroid hormone receptor), IZ-VDR (vitamin D receptor), AIZ-AR (androgen receptor), and AZ-GR (glucocorticoid receptor). Cells were incubated for 24 h with ITE-CONHCH_3_ (0.1–10 μM) either alone (agonist mode) or in combination with model receptor agonists (antagonist mode). T3, triiodothyronine; DEX, dexamethasone; DHT, dihydrotestosterone; CAL, 1α,25-dihydroxycholecalciferol. Data represent mean ± SD from three consecutive cell passages (biological replicates; n = 3). The asterisk (*) denotes a significant difference (p < 0.05) compared to vehicle control (DMSO). Statistical analyses of data coming from agonist and antagonist modes were carried out by T-test and two-way ANOVA with Dunnett’s multiple comparisons test, respectively.

### Species-selective AhR activation and AhR-ARNT heterodimerization

ITE-CONHCH_3_ acts as a potent AhR agonist but displays marked species selectivity. In reporter gene assays, the potency (EC_50_) in human intestinal cells (2.3 nM; Figure 4) and in human hepatic cells (1.6 nM (Grycova et al., 2022)) was approximately 40-fold higher than in mouse hepatic cells (75 nM; Figure 4). The ability of the compound to activate AhR was further validated by its capacity to induce AhR-ARNT heterodimerization in a cell-free system. ITE-CONHCH_3_, at concentrations of 0.1 and 1 μM, exhibited activity comparable to that of the potent AhR agonist FICZ (Figure 5).

**FIGURE 4:**
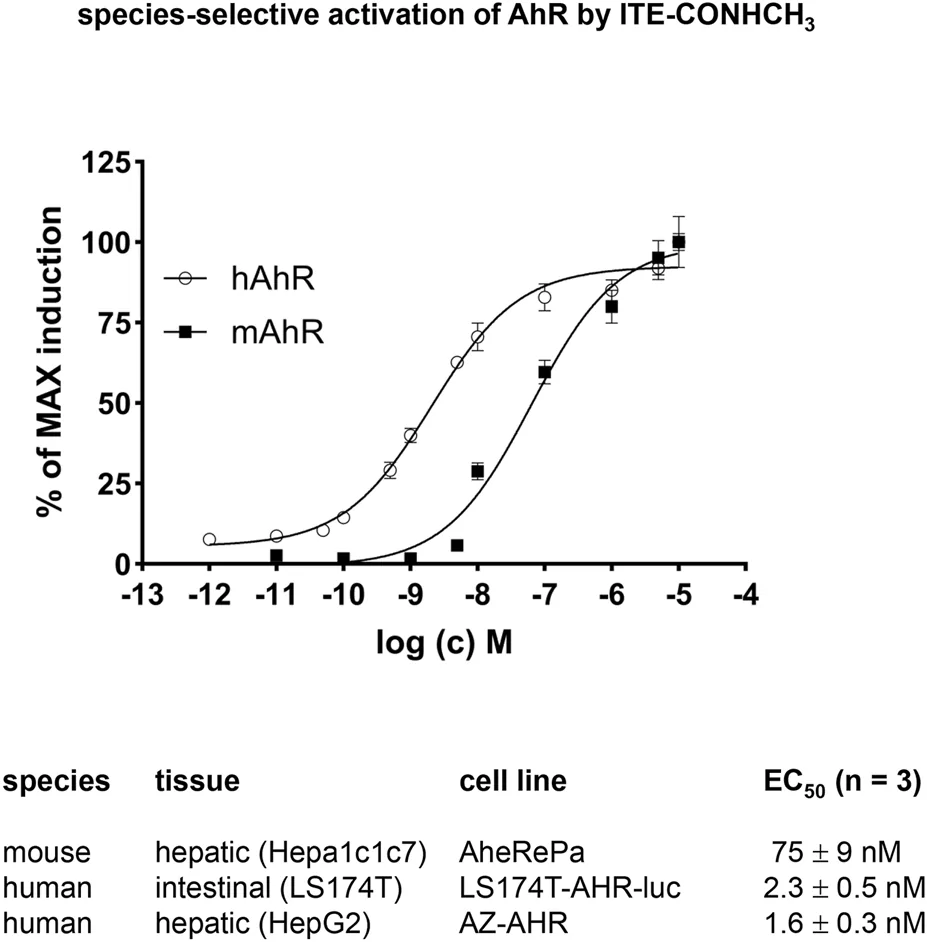
Species-selective activation of AhR by ITE-CONHCH_3_. Reporter gene assays were performed in mouse hepatic (AheRePa) and human intestinal (LS174T-AHR-luc) cells incubated for 4 h with increasing concentrations (log(c) M stands for logarithm of molar concentration) of ITE-CONHCH_3_. Data are expressed as a percentage of the maximal induction achieved in each cell line. Results are expressed as mean ± SD from three consecutive cell passages. Calculated half-maximal effective concentrations (EC_50_) are shown in the inset table.

**FIGURE 5:**
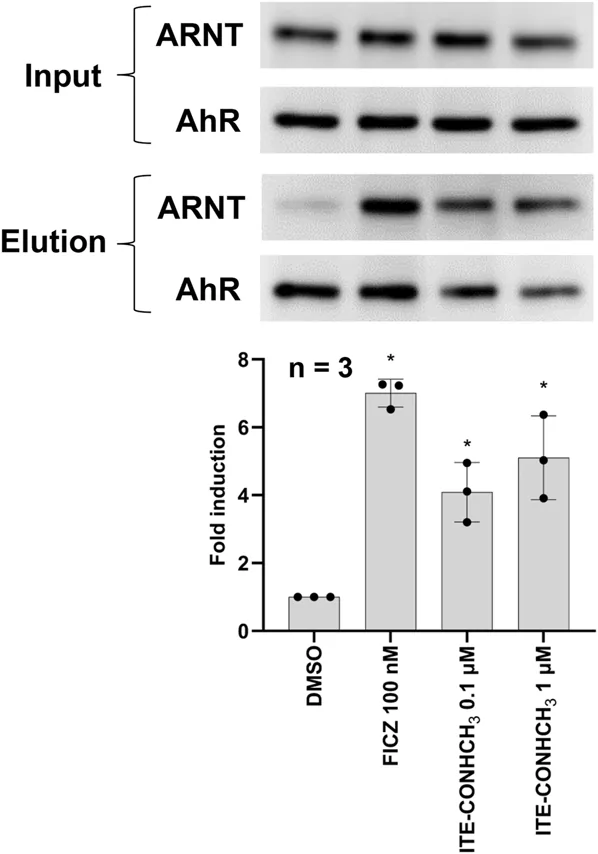
Formation of the AhR-ARNT heterocomplex in rabbit reticulocyte lysates. FL-WT-hAHR-C-6His and FL-WT-hARNT proteins were co-expressed using the TnT® Quick Coupled Transcription/Translation System in the presence of vehicle (0.1% v/v DMSO), FICZ (100 nM), or ITE-COHCH_3_ (0.1 and 1 µM), followed by His-tag purification and SDS-PAGE/Western blot analysis. Representative blots of inputs and eluates are shown. The bar graph represents the formation of the AhR-ARNT heterocomplex, shown as fold inductions over vehicle (DMSO), normalized to the corresponding AhR levels in elution samples. Data are expressed as mean ± SD from three independent experiments. The asterisk (*) denotes a significant difference (p < 0.05; one-way ANOVA with Dunnett’s multiple comparisons test) compared to vehicle control (DMSO).

### Modulation of gene expression in Caco-2 cells

In differentiated Caco-2 cells, ITE-CONHCH_3_ and the reference AhR agonist FICZ strongly induced mRNA expression of the canonical target gene *CYP1A1* and in the lesser extent the non-canonical gene *PAI-1* after 4 h of incubation (Figure 6). However, this induction was transient and decreased sharply after 24 h. This effect was more pronounced for the canonical *CYP1A1* gene. Under simulated inflammatory conditions (cytomix treatment: 20 ng/mL TNF-α, 10 ng/mL IL-1β, 10 ng/mL IFN-γ), ITE-CONHCH_3_ demonstrated anti-inflammatory potential by downregulating the mRNA levels of pro-inflammatory cytokines IL-8, IL-1β, and TNFα, showing an effect comparable to the model agonist FICZ (Figure 6).

**FIGURE 6:**
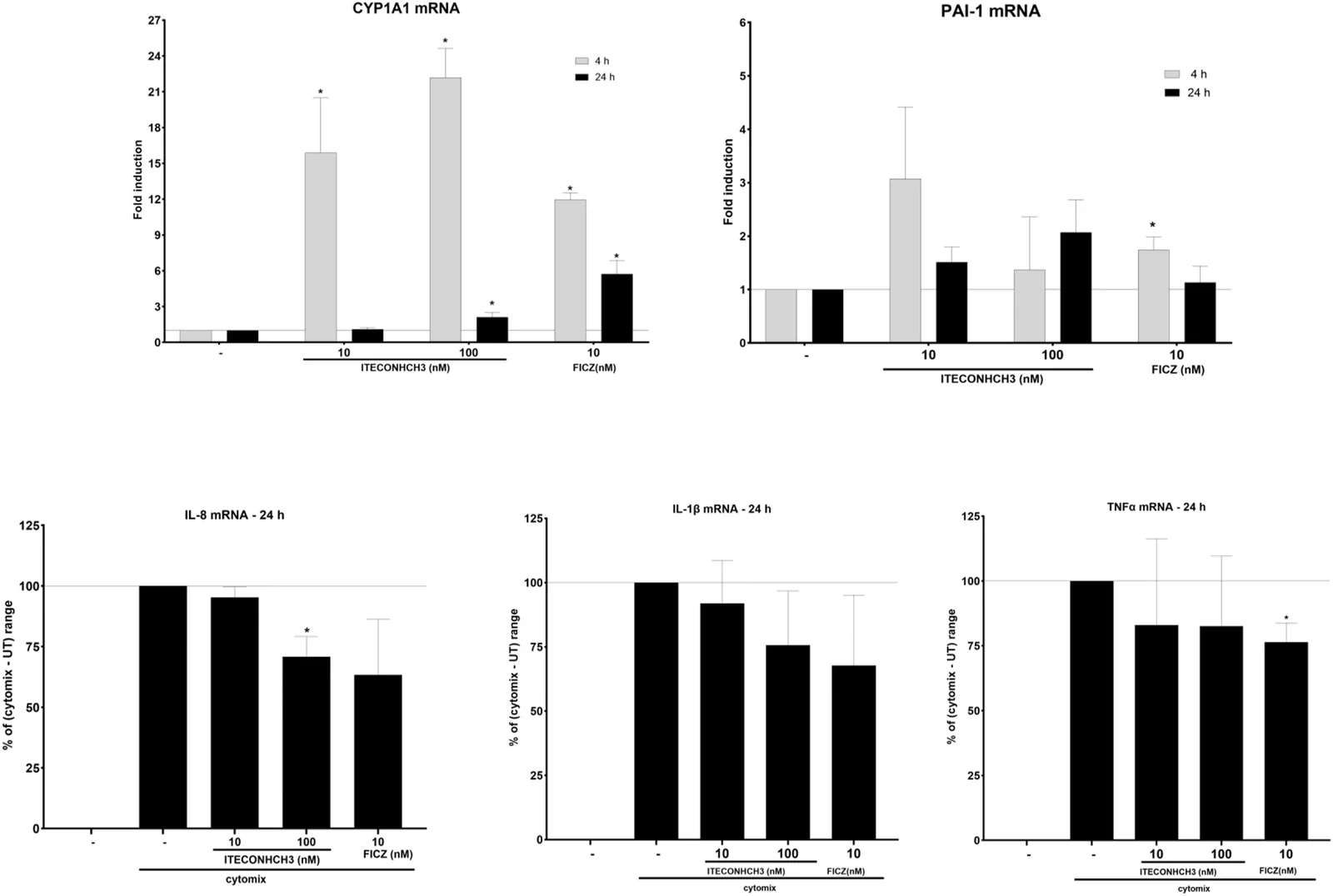
Gene expression in differentiated Caco-2 cells. Cells were treated with ITE-CONHCH_3_, FICZ or vehicle for 4 and 24 h, either alone or in combination with inflammatory cytomix. Expression levels of *CYP1A1*, *PAI-1*, *IL-8*, *IL-1β,* and *TNFα* were measured by qRT-PCR. Data represent mean ± SD from three biological replicates. The asterisk (*) denotes a significant difference (p < 0.05; two-way ANOVA with Dunnett’s multiple comparisons test) compared to the vehicle control (DMSO).

## Discussion

The development of therapeutic ligands for the AhR represents a significant frontier in modern pharmacology, particularly due to the receptor’s complex role in immune homeostasis and epithelial barrier integrity. Historically recognized as a xenobiotic sensor, the AhR is now understood as a key mediator in the cross-talk between the host and its microbiota. The concept of “microbial metabolite mimicry”—the rational design of synthetic compounds that emulate the beneficial effects of functionalized indoles produced by gut bacteria—has emerged as a promising strategy for targeting both the AhR and the pregnane X receptor (PXR) (Dvorak et al., 2020a; Dvorak et al., 2020b; Dvorak et al., 2023). In the present study, we characterized the *in vitro* pharmacological profile of ITE-CONHCH_3_, a methyl-amide bioisostere of the endogenous/microbial ligand ITE. Our findings demonstrate that this structural modification successfully addresses the critical limitation of chemical instability while preserving potent agonist activity and high receptor selectivity.

A primary barrier to the clinical translation of native ITE is its extreme metabolic fragility. Previous reports indicated a short half-life in mouse plasma due to rapid ester hydrolysis (Chen et al., 2020). Our results corroborate these findings and highlight the superiority of the amide bioisostere; ITE-CONHCH_3_ remained highly stable in both human and mouse plasma over a 120-min incubation period. This enhancement in stability is consistent with established SAR (Structure-Activity Relationship) studies of AhR-active indoles, which suggest that while the C3-substituted indole core is essential for receptor binding, the nature of the side chain considerably dictates metabolic persistence (Chen et al., 2020). The transition from an ester to a methyl-amide group provides the necessary resistance to plasma esterases without compromising the molecule’s ability to trigger the AhR signaling cascade.

A central theme in modern AhR research is the concept of functional selectivity, often framed as the “friend or foe” paradox (Dvorak et al., 2025). Unlike persistent environmental toxins such as TCDD, which cause sustained AhR activation and subsequent toxicity, microbial indoles and their mimics typically induce a transient response. In our Caco-2 cell model, ITE-CONHCH_3_ triggered a robust but transient induction of the canonical gene *CYP1A1* and the non-canonical gene *PAI-1*, with mRNA levels peaking early and declining sharply by 24 h. This “hit-and-run” activation profile is highly desirable for therapeutic applications, as it allows for the modulation of immune and barrier functions without the risks associated with chronic induction of Phase I metabolic enzymes.

The high selectivity of ITE-CONHCH_3_ was further confirmed by our off-target screening against a panel of human nuclear and steroid receptors (TR, VDR, AR, and GR). While a slight concentration-dependent decrease in AR and GR activity was noted at the highest tested concentration (10 µM), the compound showed no agonistic activity. Given that its potency for AhR is in the low nanomolar range (EC_50_ ≈ 2 nM), these minor effects at micromolar concentrations are likely negligible in a therapeutic context. Furthermore, the high intestinal permeability (P_app_ ≈ 21 × 10^−6^ cm/s) observed in the Caco-2 model, combined with the lack of active efflux, suggests favorable oral bioavailability. This is particularly relevant for the treatment of inflammatory bowel diseases, where localized delivery to the intestinal AhR is a primary goal.

One of the most striking findings of this study is the pronounced species selectivity in both potency and blood distribution. ITE-CONHCH_3_ displayed approximately 40-fold higher agonist potency in human intestinal and hepatic cells compared to mouse hepatic cells. These differences likely arise from structural variations in the ligand-binding domain of the human *versus* mouse AhR, a phenomenon well-documented in the literature for other indole-based ligands. Additionally, we identified a notable species difference in red blood cell (RBC) partitioning, with sequestration being nearly three times higher in mouse blood (47%) than in human blood (16%). These discrepancies are critical for the interpretation of preclinical data; researchers must account for the fact that a higher dose might be required in mice to achieve the same free plasma concentration and receptor activation levels expected in humans.

Mechanistically, the ability of ITE-CONHCH_3_ to promote AhR-ARNT heterodimerization in a cell-free system confirms that it acts as a direct, high-affinity ligand. This molecular interaction translates into a potent anti-inflammatory effect, as evidenced by the downregulation of pro-inflammatory cytokines (IL-8, IL-1β, and TNFα) in Caco-2 cells under simulated inflammatory conditions. This profile aligns with the behavior of “beneficial” AhR agonists that promote a regulatory immune environment (Dvorak et al., 2025).

To contextualize the pharmacological profile of ITE-CONHCH_3_ within the broader landscape of AhR agonist drug development, it is valuable to compare it with other AhR agonists that have reached clinical development or regulatory approval. Tapinarof (VTAMA®) is currently the only approved AhR agonist, having received FDA approval in 2022 for the topical treatment of plaque psoriasis, with subsequent approval for atopic dermatitis in 2024 (Keam, 2022; Silverberg et al., 2024). Tapinarof, a stilbene-derived bacterial metabolite, activates AhR with sub-nanomolar potency (EC_50_ = 0.16 nM for nuclear translocation in HaCaT keratinocytes) (Smith et al., 2017), although cross-comparison with ITE-CONHCH_3_ (EC_50_ = 1.6–2.3 nM in human reporter gene assays) is limited by the use of different assay formats. The two compounds also differ fundamentally in their therapeutic design: tapinarof is administered topically for dermatological indications, whereas ITE-CONHCH_3_ is being developed for oral delivery to target intestinal AhR in inflammatory bowel disease. Two additional AhR agonists, AT-177 and AT-193 (Azora Therapeutics; now Adial Pharmaceuticals), are currently in early clinical development. AT-177 is an oral, colon-targeted prodrug of indirubin—the most potent AhR agonist present in indigo naturalis—designed for the treatment of ulcerative colitis with minimal systemic exposure (Saiki et al., 2021). AT-193 is a topical indirubin formulation under investigation for hidradenitis suppurativa and nail psoriasis (ClinicalTrials.gov: NCT05072886, NCT04989517). Indirubin itself is a potent AhR agonist with sub-nanomolar activity (Adachi et al., 2001) and exhibits pronounced species selectivity favoring human over mouse AhR (Sladekova et al., 2023; Faber et al., 2018) - a pharmacological feature shared with ITE-CONHCH_3_. It should be noted that peer-reviewed pharmacological data specifically characterizing AT-177 and AT-193 are not yet publicly available; the available information is derived from clinical trial registries and company communications. Despite the sub-nanomolar potency of indirubin, its clinical translation has been constrained by poor aqueous solubility and the uncontrolled nature of indigo naturalis as a botanical mixture (Saiki et al., 2021). ITE-CONHCH_3_ offers a distinct approach: as a rationally designed, fully synthetic indole-thiazole bioisostere, it combines low-nanomolar AhR potency with excellent plasma stability, high intestinal permeability without active efflux, and high receptor selectivity. Calculated physicochemical descriptors further support this positioning: ITE-CONHCH_3_ exhibits a topological polar surface area (PSA) of 74.85 Å^2^ and a cLogP of 2.21, placing it within the optimal range for oral bioavailability. Its cLogP is notably lower than those of tapinarof (4.39) and indirubin (3.19) - consistent with the topical route of tapinarof and the poor aqueous solubility that has constrained indirubin’s clinical translation - while its PSA remains well within the range associated with good intestinal permeability. These properties, together with its demonstrated anti-inflammatory activity in cellular models, and anti-colitis activity in mouse model (Grycova et al., 2022) position ITE-CONHCH_3_ as a structurally distinct and pharmacologically differentiated candidate within the emerging therapeutic landscape of AhR agonists.

In conclusion, ITE-CONHCH_3_ represents an optimized microbial metabolite mimic with a favorable pharmacological profile. Its combination of high stability, selectivity, and oral permeability, coupled with its potent anti-inflammatory properties, identifies it as a superior candidate for further preclinical development. However, the observed species-specific differences in potency and blood distribution underscore the need for cautious extrapolation of mouse-derived efficacy data to human clinical settings.

## Data Availability

Source data are available with this manuscript and have also been deposited in a publicly accessible repository: https://doi.org/10.5281/zenodo.19334877.
